# Editorial: Educating the Global Workforce for Public Health

**DOI:** 10.3389/fpubh.2017.00364

**Published:** 2018-01-22

**Authors:** Sanjay P. Zodpey, Connie J. Evashwick, Michal Grivna, Roger A. Harrison, John R. Finnegan

**Affiliations:** ^1^Vice President – Academics, Public Health Foundation of India, New Delhi, India; ^2^Associate Professor, Milken Institute School of Public Health, Washington, DC, United States; ^3^Professor and Director, Institute of Public Health, College of Medicine & Health Sciences, UAE University, AlAin, United Arab Emirates; ^4^Division of Population Health, Health Services Research and Primary Care, Faculty of Biology, Medicine and Health Sciences, School of Health Sciences, University of Manchester, Manchester, United Kingdom; ^5^Professor and Dean, School of Public Health, University of Minnesota, Minneapolis, MN, United States

**Keywords:** public health education, health workforce, competencies, global health, global health workforce

**Editorial on the Research Topic**

**Educating the Global Workforce for Public Health**

A strong health workforce is one of the six building blocks of the World Health Organization’s framework for health (1). A critical mass of health professionals with sound technical knowledge is necessary to manage a health system. The education that produces a workforce of appropriate size and skills is often a crucial limiting factor in the delivery of quality health services. The health workforce is characterized by its diversity and complexity, encompassing professionals from a wide range of occupational backgrounds and types of training. The purpose of this Research Topic is to increase the shared understanding of the current status of the education of the health workforce around the globe, particularly those working in the public health sector. With this foundation, further research and evaluation studies can then be done with a perspective that addresses global workforce issues impacting access, prevention, and care.

Education has traditionally been recognized as an important tool for creating trained health professionals. However, health professionals’ education has not kept pace with the new emerging challenges, such as rapid demographic and epidemiological transitions, fresh emerging and re-emerging health challenges, new environmental and behavioral risks, and increasing appreciation of the social determinants of health. Recent globalization of health has led to international migration of health professionals, thereby leading to cross-border recognition of health workers with an appropriate skill-mix, knowledge, and competence. While some encouraging progress has been made in health workforce development globally, challenges such as investments, planning, and interventions for advancing the agenda of health workforce development continue to persist (2).

Restructured curricula and training programs are needed that emphasize primary care and prevention; basic, clinical and population sciences; evolving health-care competencies and skills; and new teaching-learning methodologies. The Lancet Commission Report (3) has also highlighted the need to develop a common strategy at a global level for postgraduate medical, nursing, and public health education that reaches beyond the confines of national borders and the silos of individual professions. This vision would require a series of instructional and institutional reforms, which should be guided by two proposed outcomes: transformative learning and interdependence in education (3).

With an aim toward advancing this agenda, this Research Topic invited manuscripts for a wide range of topics and article types. After thorough demand generation for papers, the RT received 10 submissions matching the criteria. Each article provides valuable insight into critical issues related to public health professionals’ education for global engagement.

Sawleshwarkar and Negin’s article provides an introduction to the topic with a review of the competencies for global health (Sawleshwarkar and Negin). A comprehensive literature search using relevant keywords identified 13 relevant articles for review. Three aspects of global health competency domains emerged: burden of disease and health determinants, core public health skills, and soft skills.

Frankson et al. highlighted yet another dimension of competencies for global health—the “One Health” approach. The core competency domains considered critically important for guiding curriculum development and continuing professional education using the One Health approach were identified as “management,” “communication and informatics,” “values and ethics,” “leadership,” “teams and collaboration,” “roles and responsibilities,” and “systems thinking.”

Bjegovic-Mikanovic and Otok describe the public health problems confronting public health professionals in European public health schools, the exit competencies at the end of their public health training, and the expectations of the prospective employees. Their article stresses the need for an interface between public health functions, competencies, and performance, the importance of competence-driven education, and furthermore, the need for educational institutions to look beyond national boundaries since public health challenges are increasingly global.

The article written by Pati et al. maps global health teaching in the Indian public health education context. Pati and colleagues conducted a thorough literature search of the public health programs including global health in India. They found that while this was being covered as part of larger public health programs, a distinct program for global health teaching is missing. They emphasized that more efforts need to be directed toward integrating global health into the broader public health curricula.

Negandhi et al. undertook a desk review of monitoring and evaluation (M&E) curricula and teaching in Masters programs globally, followed by a detailed review of M&E teaching across ten institutions representing four South Asian countries. The findings of the review were utilized to identify ten core competencies for M&E teaching in a consultative meeting of academicians from the four countries. The desk review showed similarities in M&E course content but variations in course structure and delivery. The identified core competencies included basic M&E concepts.

In addition to articles with a broad perspective, several articles from a single institution present case studies with lessons learned for wider application. Caron’s article focuses on support for the recommendation that all undergraduates should have access to public health education (Caron). Her paper describes the experience of the University of New Hampshire in generating an educated citizenry that is aware of comprehensive public health conflicts, thereby contributing to both a local and global perspective on learning.

Doobay-Persaud et al. conducted a survey of students seeking admission to the MSc Global Health course at the Northwestern University, as well as a separate survey of those students already active. The aim was to describe the market characteristics for this degree, provide student backgrounds that will guide curricular and programmatic improvements, and determine if these students intend to pursue degrees in global health. Results showed a wide variety of disciplines represented in students’ previous work histories, underscoring and supporting the inter-professional nature of the field and the workforce.

Kang’s article focusing on improving global health competence among nursing undergraduates echoes the significance of global public health in today’s clinical education (Kang). This article discusses the implementation and evaluation of four field training programs for undergraduates nursing students conducted in collaboration with nursing programs in developing countries.

Kershaw et al. emphasized the importance of developing health promotion skills through a self-directed project-based learning task during which the students of the medical degree program at United Arab Emirates University were introduced to public health and health promotion practice, and their soft skills such as literature searching, writing, presentation skills, and team work.

Zhao et al. presented the reforms needed in Chinese undergraduate preventive medicine programs in areas such as the traditional preventive medicine course content, revision of its curriculum structure, the need to increase practical experience, developing variety in teaching and assessment techniques, and systematic planning for curricular reforms.

Worldwide, immense focus is being placed on the public health response required to spread beyond national boundaries, and the need for the public health workforce to be appropriately armed with the knowledge, skills, and behaviors to handle these global health challenges of the 21st century. The Recife Declaration (2) has acknowledged international commitment toward building a stronger and competent health workforce for greater progress in the HRH field. The 10 articles from this Research Topic have artfully landscaped issues that directly or indirectly point toward how knowledge and skills can be imparted to public health students using innovative teaching techniques and updated curricula. Overall, this RT has stayed focused on its objective: to highlight the importance of “educating the global workforce in public health.”

## Author Contributions

All five authors contributed significantly to the Editorial. SZ took the lead in articulating the editorial and preparing the first draft. CE edited the first draft and also added other significant details in the manuscript. MG, RH, and JF also gave their respective inputs to the manuscript from time to time.

## Conflict of Interest Statement

The authors declare that the research was conducted in the absence of any commercial or financial relationships that could be construed as a potential conflict of interest.
